# Long-term outcomes of intravitreal activated protein C injection for ischemic central retinal vein occlusion: an extension trial

**DOI:** 10.1007/s00417-021-05072-9

**Published:** 2021-04-24

**Authors:** Chikako Hara, Motohiro Kamei, Hirokazu Sakaguchi, Nagakazu Matsumura, Susumu Sakimoto, Mihoko Suzuki, Kentaro Nishida, Yoko Fukushima, Kohji Nishida

**Affiliations:** 1 Department of Ophthalmology, Osaka University Graduate School of Medicine, Osaka, Japan; 2 Department of Ophthalmology, Aichi Medical University, 1-1 Yazakokarimata, Nagakute, Aichi 480-1103 Japan

**Keywords:** Activated protein C, Anti-vascular endothelial growth factor, Central retinal vein occlusion, Macular edema, Retinal nonperfusion

## Abstract

**Purpose:**

Our previous 1-year pilot study evaluated the efficacy of intravitreally injected activated protein C (APC) in 10 eyes with ischemic central retinal vein occlusion (CRVO). The reperfusion of the areas of retinal nonperfusion (RNP) exceeded 50% of the baseline in five (50%) eyes 1 year after the APC injection. The current study evaluated the long-term efficacy and safety of intravitreal APC.

**Methods:**

The 10 eyes in the pilot study were included in this study. Other treatments were administered at the physicians’ discretion after the pilot study. We evaluated visual acuity (VA), central retinal thickness (CRT) and perfusion status, and adverse events and severity over the long term.

**Results:**

The median follow-up was 60 months (range, 48–68 months). Compared with baseline, the post-treatment VA improved significantly (*P* < 0.001) from 1.39 to 1.06 logarithm of the minimum angle of resolution. The CRT improved significantly (*P* < 0.001) from 1090 to 195 μm at the last visit. The RNP areas decreased from an average 29.7 disc areas (DAs) at baseline to an average 16.5 DAs at the last examination (mean, 40 ± 6.5 months after the first APC treatment). No adverse events were related to intravitreal APC.

**Conclusion:**

No complications were associated with intravitreal APC, the clinical course improved, and improved RNP was maintained for the long term, suggesting that intravitreal APC may be an alternative treatment for CRVO.

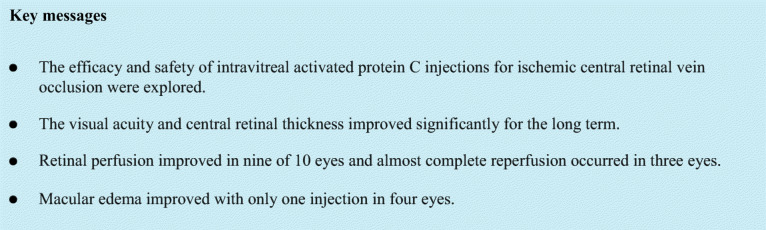

## Introduction

Retinal vein occlusion (RVO), one of the most common vascular disease, is characterized by acute presentation of intraretinal hemorrhage and macular edema (ME) associated with visual loss [1, 2]. Approximately 9% of eyes with ischemic central RVO (CRVO) develop posterior segment neovascularization, and about 40 to 60% have anterior segment neovascularization, with development of vitreous hemorrhage, retinal detachment, neovascular glaucoma, or, ultimately, total blindness [3, 4].

Treatment options for ischemic CRVO remain limited, although anti-vascular endothelial growth factor (VEGF) drugs have been approved to treat CRVO [1, 5, 6]. Recent studies, however, show that compared with branch RVO, more frequent follow-up examinations and repeated injections of anti-VEGF drugs with or without panretinal photocoagulation (PRP) are needed for CRVO due to recurrent ME in most eyes [7, 8]. Moreover, there is an unmet need for treatment options for ischemic CRVO. The Rubeosis Anti-VEgf (RAVE) study, an open-label, prospective, randomized clinical trial for ischemic CRVO, reported that nine consecutive monthly doses followed by pro re nata dosing of ranibizumab (Lucentis, Genentech Inc., South San Francisco, CA), an anti-VEGF drug, merely delayed the occurrence of rubeosis without ameliorating neovascular complications, although the retinal anatomy and vision improved [9].

Activated protein C (APC) is a regulatory enzyme involved in proteolytic inactivation of factors Va, VIIIa, and plasminogen activator inhibitor-1, which are responsible for its anticoagulatory and fibrinolytic activities [4, 10, 11]. APC also has cytoprotective, neuroprotective, anti-inflammatory, and endothelial barrier stabilization properties [12–17]. We conducted basic experiments based on the previous reports that APC protects neurons and neurovascular cells after brain ischemia [18–20] and observed that APC protected the retinal cells from ischemia in vitro and in an animal model of CRVO [21].

We then conducted a 1-year pilot study to evaluate the efficacy and safety of intravitreal APC injections in 10 eyes of nine patients with CRVO with severe ME and large areas of retinal nonperfusion (RNP). We previously reported the short-term outcomes of the first two of those cases, in which reperfusion of large ischemic areas occurred after intravitreal injection of APC [22]. Recently, the 1-year outcomes of all 10 eyes were reported, and reperfusion of the RNP areas exceeding 50% of the baseline was observed in five eyes (50%) [23]. In the current study, we report the results of an extension study that evaluated the long-term (≧ 48 months) safety and efficacy associated with the visual acuity (VA), central retinal thickness (CRT), and perfusion status of those 10 eyes treated with intravitreal APC injections for ischemic CRVO**.**

## Methods

This extension study is a retrospective study that evaluated the long-term outcomes following the 1-year clinical prospective pilot study (UMIN000008976) [23]. The Institutional Review Board approved the study protocol and the procedures conformed to the tenets of the World Medical Association’s Declaration of Helsinki. In this study, ischemic CRVO was defined as an area of retinal nonperfusion (RNP) that exceeded 10 disc areas (DAs) on fluorescein angiography (FA) images according to the Central Retinal Vein Occlusion Study [2]. All patients provided informed consent after they received an adequate explanation of the procedures to be performed. Ten eyes of nine patients who had completed the previous 1-year prospective pilot study (UMIN000008976) were included [23] and were followed for at least 36 additional months.

In the initial prospective pilot study, the study population was comprised of patients 50 years of age or older who had CRVO accompanied by ME and areas of RNP that exceeded 10 DAs on FA images. Three micrograms of human APC (Anact C®, Teijin/Chemo-Sero-Therapeutic Research Institute, Kumamoto, Japan) in a volume of 0.05 ml was injected intravitreally, and the patients were followed for 1 year. If the ME did not improve by 3 months after the first injection of APC, another APC injection was administered. When the ME recurred or no improvement of ME was observed after the second injection of APC, other treatments were administered as rescue therapy in the first year study [23]. In this extension study, intravitreal anti-VEGF drugs for recurrent ME and intravitreal anti-VEGF drugs and/or photocoagulation for complications of retinal ischemia, e.g., iris or retinal neovascularization, were administered at the discretion of the treating physicians as rescue therapy when necessary.

**Fig. 1:**
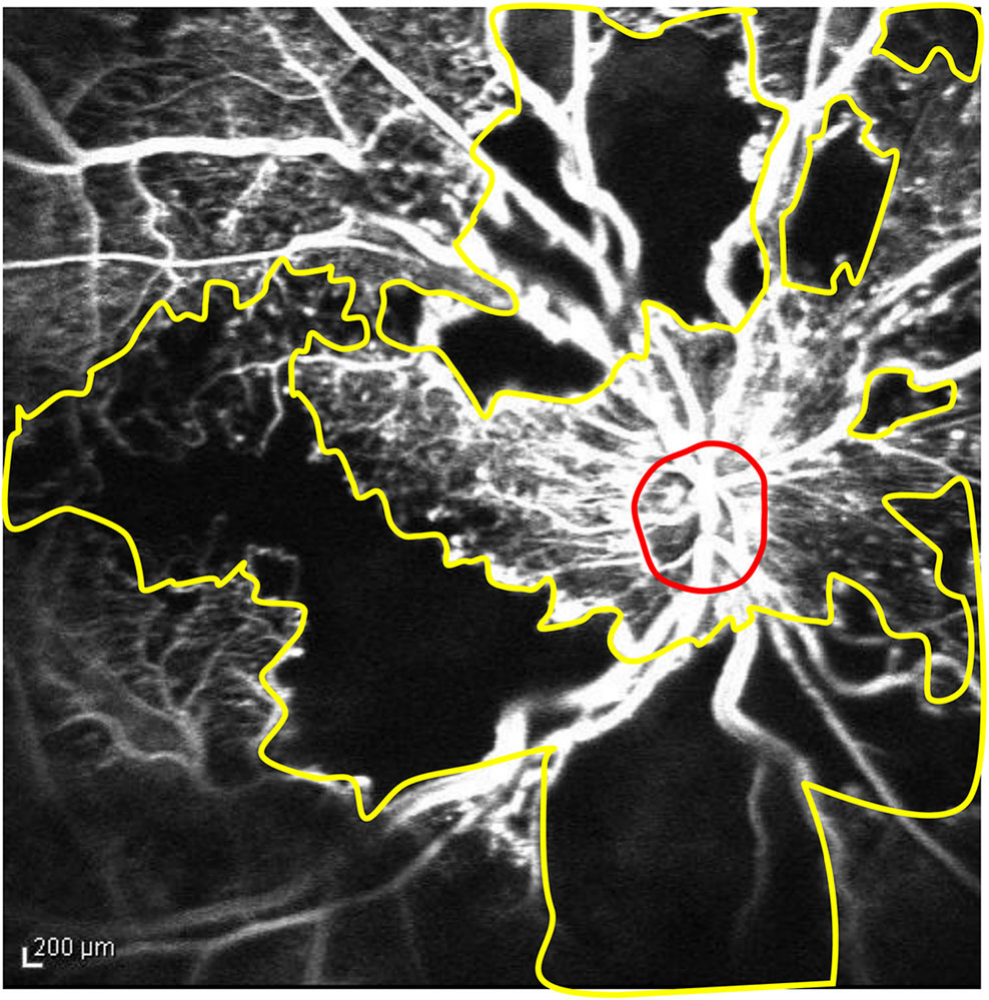
The method of evaluating the retinal nonperfusion (RNP) area. The image shows a representative macula-centered fluorescein angiography image obtained by confocal scanning laser ophthalmoscopy 30 to 35 s after fluorescein dye injection. The areas of the disc (red outline) and extensive RNP lesion (yellow outline) are, respectively, 2517 and 127,761 pixels obtained using the ImageJ software. The ratio of the RNP to the disc area (DA) in this image was calculated and the RNP area is 50.8 DAs

The main outcome measure of this study was the best-corrected visual acuity (BCVA) and the developmental rates of complications of retinal ischemia over the long term. The BCVA was measured using Landolt C charts at every study visit, and the decimal BCVAs were converted to the logarithm of the minimum angle of resolution (logMAR) value for statistical analysis. The CRT was measured at every visit using spectral-domain optical coherence tomography (Cirrus®, Carl Zeiss Meditec, Jena, Germany). Whenever possible, we performed FA annually and checked any iris or retinal neovascularization and evaluated areas of RNP as described previously [23]. A 35-degree FA image was obtained by confocal scanning laser ophthalmoscopy (HRA-2, Heidelberg Engineering Inc., Dossenheim, Germany) at baseline, 3 and 12 months, and various time points in the subsequent years at the discretion of the treating physicians. The ratio of the RNP lesion to the DA expressed in DAs is calculated using ImageJ software (National Institutes of Health, Bethesda, MD, USA) (Fig. 1).

### Statistical analysis

The differences in the BCVAs and CRTs between baseline and the post-treatment time points were evaluated using the paired *t* test. Statistical comparisons between the datasets were performed using JMP statistical software (SAS Institute, Cary, NC, USA). *P* < 0.05 was considered statistically significant.

## Results

The 10 eyes of nine patients (5 men, 4 women; mean age, 72.0 years; range, 60–78 years) with ischemic CRVO who enrolled in this initial trial were analyzed. Table 1 shows the patient characteristics.

**Table 1:** Clinical characteristics of the patients and results

Case	Sex	Age	Period of onset (weeks)	Treatment before the study	Follow-up duration (months)	Baseline and last visit						Number of APC injections (1st year)	Additional treatment		Neovascularization	
						Visual acuity (logMAR) Baseline/Last		Central retinal thickness (μm) Baseline/last		Retinal nonperfusion area disc area (% of baseline) Baseline/Last			Year 1	After year 2 (current study)	Anterior segment	Posterior segment
1	M	62	12	None	60	1.52	0.70	792	167	50.8	0 (0%)	1	None	None	–	–
2	M	74	12	IVTA+IVtPA	53	1.40	1.40	1089	144	49.7	44.8 (90%)	2	IVB*2, PC	IVB*1	+	–
3	F	75	24	IVTA+IVtPA	68	2.00	1.52	1204	190	25.1	10.5 (42%)	1	None	None	–	+
4	F	75	40	STTA, PPV IVTA +IVtPA	48	1.52	1.40	1602	172	27.3	22.2 (81%)	2	IVB*3, PC, STTA*1	None	+	–
5	F	77	21	None	60	2.00	1.52	1850	168	51.5	21.9 (41%)	3	None	None	–	+
6	M	77	3	None	60	1.05	0.30	750	212	15.8	3.85 (24%)	1	None	PC	–	+
7	M	77	2	None	53	0.82	1.00	956	336	49.9	45.7 (92%)	2	IVTA+IVtPA*2, STTA*2,IVB*3	IVB*2, IVA*4	–	–
8	F	78	12	STTA	62	1.52	1.52	990	145	11.7	0.36 (3%)	1	None	IVB*1, PC	–	+
9	M	60	3	None	60	1.22	0.40	986	189	5.3	0.36 (7%)	1	None	PC	–	+
10	M	70	12	STTA	60	0.82	0.82	687	225	10.4	15.7 (151%)	2	IVTA+IVtPA*3	None	–	–

*M* male, *F* female, *logMAR* logarithm of the minimum angle of resolution, *IVTA* intravitreal triamcinolone acetonide, *IVtPA* intravitreal tissue plasminogen activator, *STTA* subtenon triamcinolone acetonide, *PPV* pars plana vitrectomy, *APC* activated protein C, *IVB* intravitreal bevacizumab, *IVA* intravitreal aflibercept, *PC* photocoagulation

At baseline, the mean duration from symptom onset to the first injection of intravitreal APC was 14.1 weeks (range, 2–40 weeks). Seven (70%) of 10 eyes were treated first with an intravitreal APC injection by 12 weeks after symptom onset. The median period of follow-up was 60 months (mean, 57.9; range, 48–68 months).

### BCVA

**Fig. 2:**
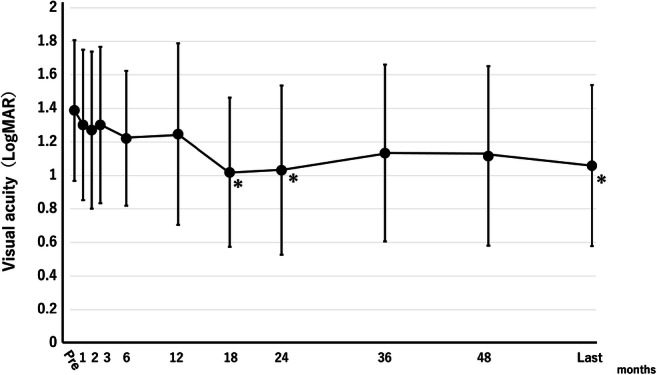
The changes in the best-corrected visual acuity (BCVA) before and after treatment with activated protein C. The graph shows the mean BCVA from baseline to the last visit. The mean BCVA improved significantly (*P < 0.05) from 1.39 logarithm of the minimum angle of resolution (logMAR) at baseline to 1.02, 1.03, and 1.06 logMAR at 18 and 24 months and the last visit, respectively. The BCVA improved gradually, but the difference did not reach significance until 12 months

The BCVA values are 1.39 ± 0.42, 1.25 ± 0.54, 1.02 ± 0.44, 1.03 ± 0.50, 1.13 ± 0.53, 1.12 ± 0.53, and 1.06 ± 0.48 logMAR at baseline, 12, 18, 24, 36, and 48 months, and last visit, respectively (Fig. 2). Statistical significance compared with baseline was reached at 18 and 24 months and the last visit (*P =* 0.01, 0.02, and 0.02, respectively), although the differences did not reach significance at 12, 36, and 48 months (*P* = 0.36, 0.09, and 0.10, respectively) because of variations in the values. Five (50%) of 10 eyes ultimately had a visual improvement of three lines or more (Table 1).

### ME

**Fig. 3:**
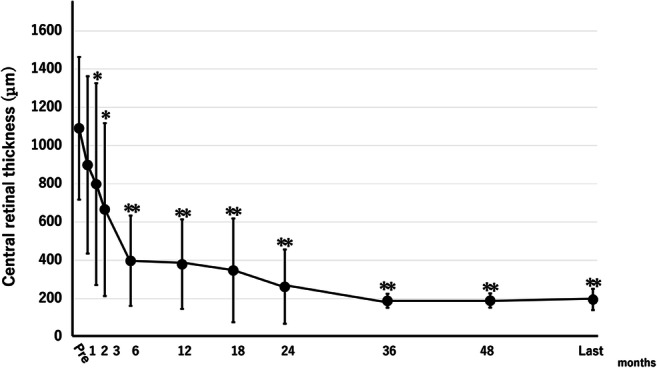
The central retinal thickness (CRT) before and after treatment with activated protein C. The graph shows the mean CRT from baseline to the last visit. The mean CRT improved gradually and significantly (*P* < 0.0001) from 1091 μm at baseline to 798 and 195 μm at 2 months and the last visit, respectively

At baseline, all eyes had severe ME with a mean CRT of 1091 ± 374 μm, which improved significantly (*P* < 0.0001) from 2 months after the first injection onward (Fig. 3). In five eyes (cases 1, 3, 6, 8, and 9), the ME resolved completely after the first injection of APC. In four (cases 1, 3, 6, and 9) of the five eyes, the ME did not recur over the study course with no other additional treatment. In one (case 8), the ME resolved completely after the first intravitreal injection of APC and remained stable for 12 months, after which the ME recurred minimally only at 14 months after the initial APC injection; the ME resolved completely again with an injection of 1.25 mg of bevacizumab (Avastin, Genentech Inc.) and has not recurred for more than 3 years of follow-up after the rescue treatment. In case 5, the CRT decreased, but residual ME remained after the first APC injection. Administration of two additional injections of APC (2 and 6 months after the first injection) resulted in complete resolution of the ME, which has not recurred during 5 years of follow-up. In the remaining four eyes (cases 2, 4, 7, and 10), the CRT did not change during the 3 months after the APC injections, and additional rescue treatments (intravitreal anti-VEGF drugs, sub-Tenon triamcinolone acetonide, and intravitreal tissue plasminogen activator) are administered (Table 1). Ultimately, the ME resolved completely in all 10 eyes and the CRT was less than 200 μm.

### Area of RNP in the posterior pole

**Fig. 4:**
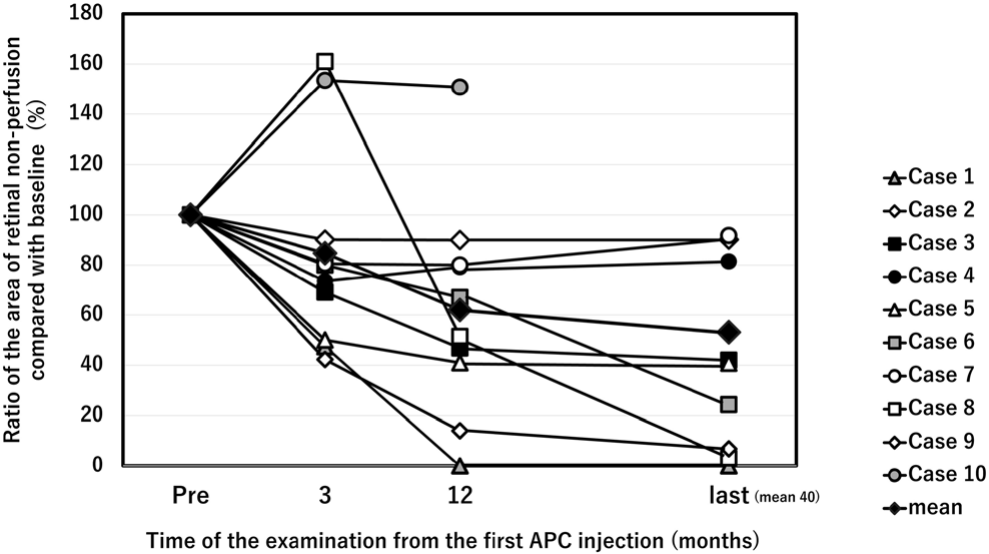
The time course of the retinal nonperfusion (RNP) area. In six eyes (cases 1, 3, 5, 6, 8, and 9), the area of RNP improved to less than 50% of the baseline area at the last examination, and almost complete reperfusion occurred in three of the eyes (cases 1, 8, and 9) (0 to 7% of the baseline RNP area remains). In the remaining four eyes, the RNP area decreased in three eyes (cases 2, 4, and 7) to some degree (81–92% of baseline). Only one eye (case 10) had a moderate increase in the RNP area (151% of baseline) 1 year after the treatment and that change could not be evaluated because the patient refused additional fluorescein angiography. APC = activated protein C

**Fig. 5:**
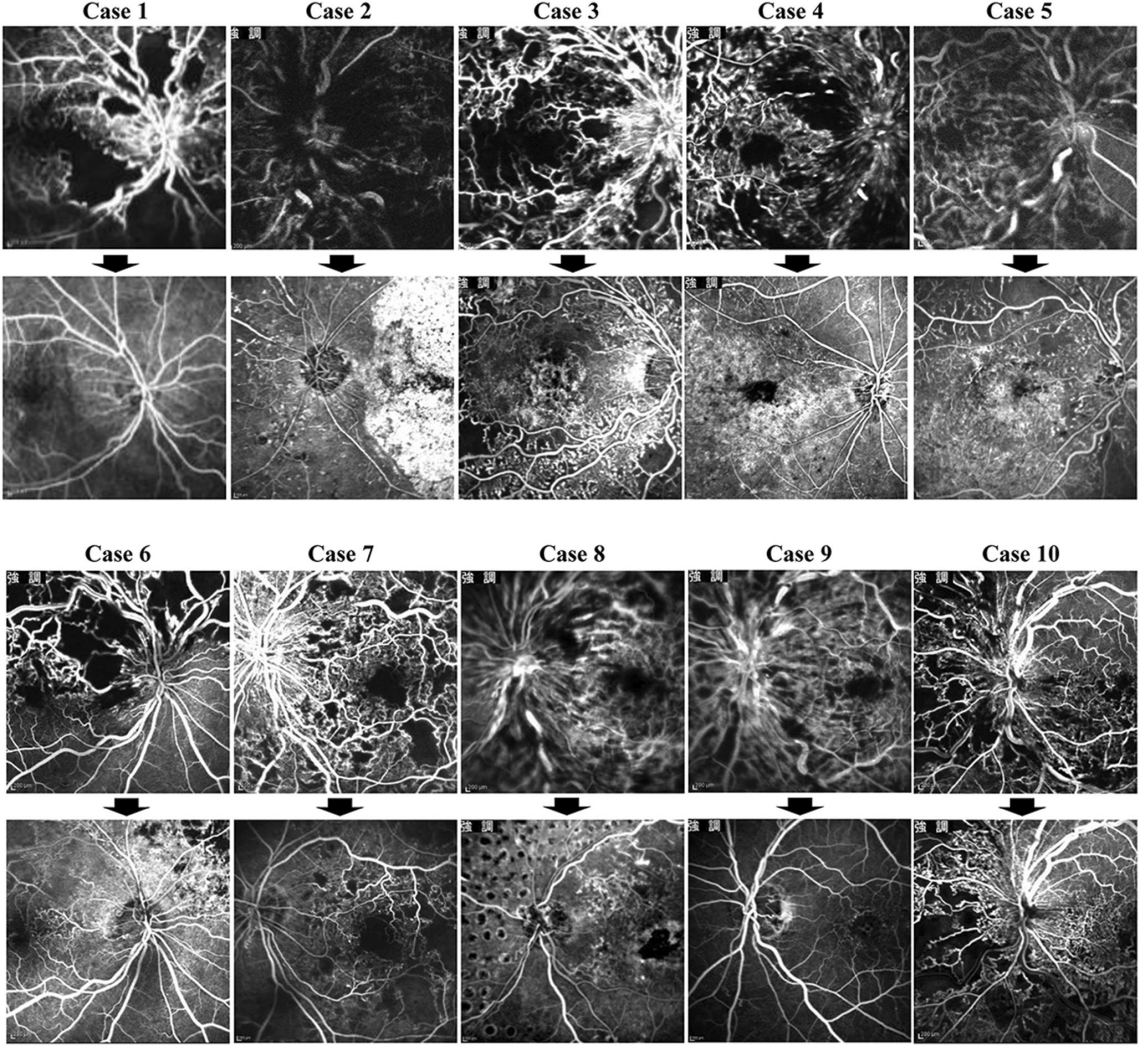
The perfusion status evaluated by fluorescein angiography performed before and after treatment. The top row shows the pretreatment status; the bottom row shows the post-treatment status. Six eyes (cases 1, 3, 5, 6, 8, and 9) have marked reperfusion. Three eyes (cases 1, 8, and 9) especially have almost complete reperfusion. Three eyes (cases 2, 4, and 7) have minimal reperfusion, and one eye (case 10) has a moderate increase in the retinal nonperfusion area

At baseline, the mean area of RNP in the posterior pole seen on FA was 29.7 ± 19.0 DAs and significantly (*P* = 0.020) decreased to 17.8 ± 16.2 DAs at the end of the initial 1-year study [23]. In one eye (case 10), the patient refused additional FA after the first year. In the other nine eyes, additional FA was performed at a mean of 40 ± 6.5 months after the first APC injection. At the last examination, the RNP significantly (*P* = 0.015) decreased to 16.5 ± 17.3 DAs. In six (60%) eyes (cases 1, 3, 5, 6, 8, and 9), the areas of RNP decreased to 50% or less of those at baseline. These six eyes were defines as responders. In three eyes (cases 1, 8, and 9) among these six responders, the entire area of RNP is virtually reperfused, and only 0 to 7% relative to the baseline area of RNP remains nonperfused (Figs. 4, 5). However, there was no definite improvement in perfusion in the other four eyes that were nonresponders (cases 2, 4, 7, and 10). In three of the four nonresponders (cases 2, 4, and 7), the area of RNP decreased by 81 to 92% of the baseline value. Only one eye (case 10) had an increase in the RNP area (151% of baseline) at the end of the initial 1-year study, and the subsequent change could not be evaluated because the patient refused additional FA. In the remaining nine eyes, after the first year, the RNP decreased further in two eyes (cases 6 and 8), and the retinal ischemia did not worsen in any eyes (Figs. 4, 5 and Table 1).

A comparison of the responder and nonresponder groups showed that the baseline areas of RNP and the timing of the initial treatment did not differ significantly; the mean areas of RNP at baseline in eyes with and without a profound effect were, respectively, 21.7 vs. 28.4 DAs (*P* = 0.60), and the periods between the onset and the first APC treatment were, respectively, 10.8 vs. 14.8 weeks (*P* = 0.41).

In six eyes (cases 1, 3, 5, 6, 8, and 9) in which the ME almost improved with only APC injection, the areas of RNP also decreased to 50% or less of those at baseline. In other words, in the remaining four eyes (cases 2, 4, 7, and 10), neither ME nor RNP improved with only APC treatment.

### Development of neovascular complications

During the first year, anterior segment neovascularization developed in two (20%) eyes (cases 2 and 4), and posterior segment neovascularization developed in four (40%) eyes (cases 3, 6, 8, and 9). During the follow-up period after the second year, posterior segment neovascularization newly developed in one eye (case 5). Neovascularization in both ocular locations did not develop simultaneously in any eyes. In the two eyes (cases 2 and 4) with anterior neovascularization, rubeosis was detected 4 and 10 months after the first APC treatment. In one of those eyes (case 4), PRP combining with an intravitreal bevacizumab injection resulted in decreased neovascularization, and the elevated intraocular pressure (IOP) returned to the normal range. In the other eye (case 2), the IOP did not return to the normal range despite the same treatments, and trabeculectomy was performed to control the IOP. Of the five eyes in which posterior neovascularization developed, two eyes (cases 3 and 5) are only observed, since the neovascularization was subtle; the other three eyes (cases 6, 8, and 9) are treated with PRP, which caused regression of the neovascularization (Table 1). Of six responders (cases 1, 3, 5, 6, 8, and 9), five eyes (cases 3, 5, 6, 8, and 9) developed posterior neovascularization, and no eyes developed anterior neovascularization. In contrast, in four nonresponders (cases 2, 4, 7, and 10), no eyes developed posterior neovascularization, and two eyes (cases 2 and 4) developed anterior neovascularization. In two eyes (cases 8 and 9), neovascularization developed despite the almost completely improvement of RNP. The reason for this is thought to be that not all RNP in the peripheral area improved because of evaluation of the RNP in only the posterior retina.

### Adverse events and safety

The intravitreal injections of APC were well-tolerated, and slit-lamp and fundus examinations, Goldmann perimetry, and electroretinography showed no ocular adverse events, i.e., endophthalmitis, retinal detachment, traumatic cataract, or intraocular inflammation. Systemic adverse events also did not occur during the follow-up period.

## Discussion

In the current study, we reviewed the long-term outcomes of intravitreal APC injection for patients with ischemic CRVO. The mean VA and CRT improved significantly over the long term after the intravitreal APC injections, and apparent reperfusion of the RNP occurred in 60% of the eyes. During the long term, no ocular or systemic adverse events occurred.

Recently, the RAVE study reported the results of intravitreal injections of ranibizumab for eyes with ischemic CRVO [9]. When we compared the results of the current study with those of the RAVE study, the improvements in the VA and CRT were equivalent, i.e., from 1.39 to 1.06 logMAR VA and from 1091 to195 μm of CRT in the current study compared with from 15 Early Treatment Diabetic Retinopathy Study letters to 36.4 letters (1.40 to 0.97 in logMAR VA) and from 485 to 203 μm in the RAVE study. Regarding the development of neovascular complications, posterior segment neovascularization occurred in 33% of eyes and anterior segment neovascularization in 28% in the RAVE study, while, in the current study, the respective values were 50% (subtle 20%, apparent 30%) and 20%, which also were comparable. We hypothesized that the reasons why the incidence of neovascular complications did not decrease despite reperfusion in response to APC injections in some eyes were that APC was likely to have less or no neovascularization-suppressing activity and might even promote vascularization and that initiation of neovascularization might occur earlier than completion of reperfusion, which occurred gradually and progressed throughout the first 1 or 2 years after the APC injection (Fig. 3). These speculations that neovascularization might occur earlier than completion of reperfusion are supported by the natural history of ischemic CRVO, in that 70% of neovascular complications occurred within 3 months after onset and almost all by 9 months [2].

In contrast to the equivalent improvements in vision and incidence of neovascular complications, the numbers of treatments seem considerably different, although we cannot directly compare the two studies with different settings. A mean of 17.2 injections was administered during the 36-month RAVE trial and only 1.6 injections of APC and 2.0 intravitreal injections of other drugs during the same period in the current study, although there were some differences in the study protocols.

Importantly, reperfusion of more than 50% of the areas of RNP occurred in 60% of the current patients. The mean degree of improvement in the RNP resulting from an APC injection was as large as 15.3 DAs in the nine eyes with any improved perfusion. We are encouraged by these results given that the area of RNP will not improve spontaneously or may even progress in eyes with ischemic CRVO during the natural disease course [24, 25] and during the course of anti-VEGF therapy [26]. Frequent injections of an anti-VEGF drug also have been reported to promote improved or reduced progression of RNP [27, 28] but not in long-term observation with as-needed injections [28], and especially in CRVO, the area of nonperfusion increased with time. The current results support the potential for intravitreal injections of APC to promote long-term reperfusion of large areas of RNP. While the mechanism of improvement in RNP after intravitreal APC injections is not understood completely, the striking improvement in reperfusion and visual function in the eyes of patients with ischemia merits further investigation.

Interestingly, most eyes either exhibited a profound effect or no effect on both the ME and RNP. When we divided the 10 eyes into the responders (cases 1, 3, 5, 6, 8, and 9) and nonresponders (cases 2, 4, 7, and 10), the factors predictive of good clinical responses were unclear. The areas of RNP at baseline or the timing of the initial treatment did not differ significantly between the responder and nonresponder groups.

The mechanism of improvement of the ME and RNP by intravitreal APC injection also is unknown. APC is a coagulation regulatory factor and has anticoagulant activities and fibrinolytic activity [4, 10, 11]. These anticoagulant/fibrinolytic effects contribute to prevention of further obstruction and resolution of coagulation in the vein and capillaries. It also has been reported that APC can stabilize the vessels [29, 30]. These activities might reduce edema but not reperfuse the retinal vessels. If the improved retinal circulation resulted from the direct anticoagulant/fibrinolytic and vascular stabilizing activities, the reperfusion likely would occur more rapidly after the injections, while the reperfusion occurred gradually and progressed throughout 1 or 2 years in the current study. APC has been reported to provide neuroprotection during transient ischemia and promote activation of anti-apoptotic mechanisms in brain cells by acting directly on the endothelium and neurons [10, 12, 16, 17, 21, 27, 28]. Our previous experiments showed that APC protected retinal cells from apoptosis under hypoxic conditions [21]. Therefore, we speculated that reperfusion associated with APC might occur by promoting survival of retinal and vascular cells and proceed via neurovascular cross-talk.

This study is limited by its small sample size. A larger clinical trial is needed. Another limitation of this study was that the retinal perfusion status was evaluated only in the posterior retina. Evaluation using ultra-wide-field FA images would be better, but we did not have an ultra-wide-field FA camera at the beginning of the initial study. Evaluation of the perfusion in the posterior retina provides limited information but might be acceptable, because Mir et al. [28] compared the grading of the RNP centrally with that in the periphery and found a significant correlation between them. In conclusion, the current results suggested that APC might produce prolonged reperfusion of large areas of RNP, improve VA and ME, and not be associated with severe complications in eyes with ischemic CRVO. The results may be informative when considering alternative treatments for ischemic CRVO. Further investigation of APC therapy is warranted not only for CRVO but also for other ischemic retinal diseases.
